# Dopamine D2/3- and μ-opioid receptor antagonists reduce cue-induced responding and reward impulsivity in humans

**DOI:** 10.1038/tp.2016.113

**Published:** 2016-07-05

**Authors:** S C Weber, B Beck-Schimmer, M-E Kajdi, D Müller, P N Tobler, B B Quednow

**Affiliations:** 1 Department of Economics, Laboratory for Social and Neural Systems Research, University of Zurich, Zurich, Switzerland; 2 Institute of Anesthesiology, University Hospital Zurich, Zurich, Switzerland; 3 Institute of Clinical Chemistry, University Hospital Zurich, Zurich, Switzerland; 4 Neuroscience Center Zurich, University of Zurich, Swiss Federal Institute of Technology Zurich, Zurich, Switzerland; 5 Experimental and Clinical Pharmacopsychology, Department of Psychiatry, Psychotherapy and Psychosomatics, Psychiatric Hospital, University of Zurich, Zurich, Switzerland

## Abstract

Increased responding to drug-associated stimuli (cue reactivity) and an inability to tolerate delayed gratification (reward impulsivity) have been implicated in the development and maintenance of drug addiction. Whereas data from animal studies suggest that both the dopamine and opioid system are involved in these two reward-related processes, their role in humans is less clear. Moreover, dopaminergic and opioidergic drugs have not been directly compared with regard to these functions, even though a deeper understanding of the underlying mechanisms might inform the development of specific treatments for elevated cue reactivity and reward impulsivity. In a randomized, double-blind, between-subject design we administered the selective dopamine D2/D3 receptor antagonist amisulpride (400 mg, *n*=41), the unspecific opioid receptor antagonist naltrexone (50 mg, *n*=40) or placebo (*n*=40) to healthy humans and measured cue-induced responding with a Pavlovian-instrumental transfer task and reward impulsivity with a delay discounting task. Mood was assessed using a visual analogue scale. Compared with placebo, amisulpride significantly suppressed cue-induced responding and reward impulsivity. The effects of naltrexone were similar, although less pronounced. Both amisulpride and naltrexone decreased average mood ratings compared with placebo. Our results demonstrate that a selective blockade of dopamine D2/D3 receptors reduces cue-induced responding and reward impulsivity in healthy humans. Antagonizing μ-opioid receptors has similar effects for cue-induced responding and to a lesser extent for reward impulsivity.

## Introduction

Substance addiction is characterized by uncontrolled drug use, drug craving and a high incidence of relapse even after years of abstinence. Cue reactivity and reward impulsivity are two core features of addiction that have an important role in the development and maintenance of drug addiction as well as relapse.^1^ Cue reactivity refers to the ability of drug-associated stimuli to increase responding to those drug cues in addiction. It is often used to explain why patients with addiction use drugs and relapse at a higher rate in environments that have been associated with prior drug use. Objects and environments that are paired with drug use become conditioned stimuli capable of independently triggering instrumental drug-seeking behaviors.^1^ Not surprisingly, elevated cue reactivity is consistently found in substance-use disorders.^2, 3^ Reward impulsivity is defined as the inability to delay gratification and wait for a larger reward, in the face of a smaller immediate reward.^4^ Increased reward impulsivity has been suggested as a stable marker (endophenotype) of addiction^4, 5, 6, 7^ and may explain the reduced ability of affected individuals to refrain from taking drugs even when continued use is associated with high personal and financial costs.

As both cue reactivity and reward impulsivity are important factors in drug addiction, understanding their underlying neurochemistry may provide key insights into drug abuse and relapse. Two neurotransmitter systems have been particularly implicated in addiction—the dopamine and the opioid systems.^8^ Opioid receptor agonists and antagonists are commonly prescribed to reduce craving and to prevent relapse in opioid dependence and other forms of substance addiction.^9^ On the other hand, in animal models, most addictive drugs increase dopamine levels in the nucleus accumbens,^10^ which has been confirmed in humans for stimulant drugs, alcohol and nicotine.^11^ Moreover, stimulant-addicted individuals show a blunted dopamine response to acute challenges with stimulants, but increased dopamine release in response to sensory cues associated with drug use.^12^ It is therefore of high interest to understand how cue reactivity and reward impulsivity are commonly and differentially influenced by dopamine and opioid blockade.

Here, we investigate the pharmacological basis of cue reactivity and reward impulsivity in healthy volunteers. The use of healthy volunteers to study how reward processing may be altered in addiction offers several important benefits. First, it makes human studies comparable to the numerous animal studies that mainly use pharmacological manipulations on healthy animal subjects. Second, using healthy volunteers makes it easier to interpret the results of the pharmacological intervention, as it dissociates drug effects from disorder effects and is not complicated by interactions between drug and disorder. Third, patients with substance-use disorders often have comorbidities and are treated with psychotropic medications that potentially interact with experimental drug challenge effects. In the current study, we probe the effect of dopamine and opioid receptor antagonists in a Pavlovian-instrumental transfer (PIT) task and a delay-discounting task. PIT is a common measure of cue-induced responding (cue reactivity) that has been used in numerous animal studies and has also been applied to humans.^13^ It measures the ability of a previously rewarded conditioned stimulus to trigger instrumental responding even in the absence of any rewards. PIT tasks usually employ a three-phase design: in an instrumental and a Pavlovian phase, an instrumental response to earn reward is acquired and a Pavlovian conditioned stimulus predicting reward is learned. During the critical test phase, which measures PIT/cue-induced responding, the conditioned stimulus is displayed in the absence of rewards and instrumental responding is recorded. The ability of the conditioned stimulus to elicit instrumental responding during the test phase is considered a model of how drug-associated stimuli can trigger drug-seeking behavior.^14^ Reward impulsivity is often measured using delay-discounting tasks.^4, 15, 16^ In these tasks, participants choose between smaller immediate rewards and larger delayed rewards, and reward impulsivity is characterized by an increased preference for smaller immediate rewards over larger delayed rewards, that is, higher discounting.^4, 15, 16^

In separate studies, PIT and delay discounting have been linked to the dopamine system (delay discounting: for example 17, 18, 19; PIT: for example 20, 21, 22, 23, 24) and the opioid system (delay discounting: for example 25, 26, 27; PIT: for example 28, 29). However, the previous results are primarily from animal studies (for a non-exhaustive overview, see Table 1) and often contradictory because various and relatively unselective challenge drugs have been used. In addition, the rare human studies (Table 1) have mostly tested rather small samples. More importantly, no study directly compared dopaminergic and opioidergic drug challenges on reward impulsivity and cue-induced responding.

To fill this gap, we investigated the role of the dopamine and opioid systems in cue-induced responding and reward impulsivity by administering the highly selective D2/D3 receptor antagonist amisulpride, the non-selective opioid antagonist naltrexone and placebo in a randomized, double-blind, between-subject design in healthy volunteers. We used 400 mg amisulpride and 50 mg naltrexone administered orally, a standard dosage with only minor side effects in several previous studies.^40, 41^

## Materials and methods

### Participants

A total of 121 healthy volunteers, recruited from the *Laboratory for Social and Neural Systems Research* subject pool, participated in the study. The sample size was chosen based on previous literature and in order to obtain a statistical power of 80% for detecting significant differences between drug conditions.^41^ All participants were screened by the recruitment team to ensure that they were physically and psychiatrically healthy. Specific exclusion criteria were a history of brain disease or injury, surgery to head or heart, neurological or psychiatric diseases (including alcoholism, depression, schizophrenia, bipolar disorders, claustrophobia or Parkinson symptoms), a severe medical disease such as diabetes, cancer, insufficiency of liver or kidneys, acute hepatitis, high or low blood pressure, any cardiovascular incidences, epilepsy, pregnancy or breastfeeding, past use of opiates or other drugs that may interact with amisulpride or naltrexone (such as stimulants). Illegal drug use (amphetamines, barbiturates, buprenorphine, benzodiazepines, cannabis, cocaine, MDMA, methadone and morphine/opiates) was controlled by drug urine testing (M-10/5-DT, Diagnostik Nord, Schwerin, Germany) and cardiac health was confirmed by electrocardiogram. All participants provided written informed consent. The study was approved by the ethics committee of the canton of Zurich and registered on www.clinicaltrials.gov (NCT02557984).

### Procedure

On average, 3 h (±1.10 min, s.e.m.) before the experimental tasks, participants received a pill containing either placebo (*N*=40), 400 mg amisulpride (*N*=41) or 50 mg naltrexone (*N*=40) in a randomized and double-blind manner (Supplementary Figure S1). Randomization was performed in blocks of nine participants by the study pharmacist. Amisulpride is a selective dopamine D2/D3 receptor antagonist, whereas naltrexone is an unspecific opioid receptor antagonist that acts primarily on the μ- and κ-opioid receptors, with lesser and more variable effects on δ-opioid receptors.^41, 42^ The two active doses were chosen to result in comparable neurochemical responses. Whereas 400 mg amisulpride usually result in ~50–80% D2 receptor occupancy,^43, 44, 45, 46^ 50 mg naltrexone normally cause >90% mu-opioid receptor occupancy.^42, 47^ As D2 receptor occupancies of >90% are only attainable with amisulpride doses of 800 mg or higher,^43, 45, 46^ we nevertheless decided to compare 400 mg amisulpride and 50 mg naltrexone—doses that are both well tolerated in healthy subjects^40, 41^—in order to avoid extrapyramidal side effects potentially associated with higher amisulpride doses. To enhance and equate absorption time across participants, all participants were asked not to eat for 6 h before arrival. After task completion, participants answered post-experimental questionnaires, which probed whether they thought they had received a drug or placebo, and also measured their mood (one rating was not recorded in the placebo group). Using high-performance liquid chromatography–mass spectrometry, amisulpride and naltrexone blood plasma levels immediately before and after the behavioral tasks were determined in order to control for absorption of the drugs (amisulpride before: 618 μg l^−1^, after: 915 μg l^−1^, mean: 767 μg l^−1^; naltrexone before: 2.98 μg l^−1^, after: 2.50 μg l^−1^, mean: 2.74 μg l^−1^). There was no correlation between the blood plasma level and task performance (*PIT*: |r|<0.20, *P*>0.24, *N*_amisulpride_=35, *N*_naltrexone_=34; *DD*: |r|<0.15, *P*>0.36, *N*_amisulpride_=40, *N*_naltrexone_=40).

### PIT task

The PIT task (duration: 23.46 min±0.42) followed the standard three-phase PIT design (please refer to the supplement for a more detailed description) according to the protocol of Lovibond and Colagiuri.^48^ Initially, in the instrumental conditioning phase, participants needed to press a button in order to earn a chocolate M&M's reward on a variable-ratio 10 schedule. Subsequently, in the Pavlovian phase, a differential-conditioning procedure was used in which an appetitively conditioned stimulus (CS+) was always paired with the delivery of a chocolate M&M's reward, whereas a neutral stimulus (CS-) was always presented with no outcome. Lastly, participants completed the transfer-test phase, where no rewards were available. Both the CS+ and the CS- were presented twice for 10 s in random order, while button presses were recorded (Supplementary Figure S2). Before and after the task, participants were asked to indicate their desire for M&M's in order to control for hunger levels. Using the same standard as in the previous study,^48^ two placebo, six amisulpride and six naltrexone participants did not meet the criterion of the instrumental phase and were therefore excluded from the PIT analysis. For an overview of excluded subjects for each task, please refer to Supplementary Table S1.

### Delay-discounting task

After the PIT task, participants completed the Kirby (1999) Monetary Choice Questionnaire^15^ to measure delay discounting (duration: 1.8 min±0.04). The questionnaire consisted of 27 hypothetical decisions in which participants chose between a smaller, immediate monetary reward and a larger, delayed monetary reward. It included nine questions for each of three delayed reward magnitudes (small, medium and large). The monetary rewards varied between 11 Swiss Frank (CHF) and 80 CHF for immediate rewards, and between 25 CHF and 85 CHF for delayed rewards. The delays of the delayed reward varied between 7 and 186 days. One female subject in the amisulpride group did not complete the delay-discounting task and was therefore excluded from all analyses of this task.

### Assessment of affect, mood and trait impulsivity

Before drug administration, participants completed the Barratt Impulsiveness Scale (BIS-11)^49^ in order to measure trait impulsivity, the short version of the Action Regulating Emotion Systems questionnaire^50^ to check for differences in the Behavioral Inhibition and the Behavioral Activation System scales (BIS/BAS), as well as the Affect Intensity Measure^51^ to assess affective responsiveness.

After the behavioral tasks, participants rated their current mood on the computer using a visual analogue scale that ranged from 0 (very bad mood) to 100 (very good mood). They were instructed to ‘please mark on the scale how you feel right now.'

### Statistical analysis

To assess whether our groups differed in age, body mass index, years of education, trait impulsivity, BIS/BAS score and affect intensity, we conducted one-way analyses of variance (ANOVAs) with these measures. In addition, we performed a *X*^2^-analysis of whether the subjects correctly guessed whether they received a medication or placebo.

In order to assess Pavlovian and instrumental learning, we analyzed the performance of the groups in the first two phases of the PIT task, using one-way ANOVAs with the between-subject factor drug group. Specifically, we compared the number and frequency of button presses, the time participants took to reach the criterion for the instrumental phase and the ratings of the reward contingencies for the Pavlovian phase. For the main analysis of interest, we focused on differences in the number of button presses during the transfer-test phase. We normalized the button presses during the CS test phase by the number of responses during the initial extinction period of the transfer-test phase. However, the results did not change when the raw (non-normalized) data were used and the groups did not differ significantly in button-pressing during the extinction period (Supplementary Table S2). In order to probe the cue-related increase in instrumental responding, we compared button presses during the 10-s CS presentation with the button presses in the 10 s before the CS presentation for CS+ versus CS−. We performed a mixed-model ANOVA to compare the two drug groups with the placebo group, with group as the between-subject factor and CS type and time as the within-subject factors. Significant findings (*P*<0.05) were followed by *post hoc t*-test analyses.

For the delay-discounting task we measured how often participants chose the smaller immediate reward, as opposed to the larger delayed reward to estimate reward discounting. More frequent choice of immediate rewards corresponds to stronger discounting. This use of the proportion of immediate rewards chosen allowed us to analyze the discounting behavior without relying on assumptions about the shape of the discounting curve for the individual participants.^52^ However, using Kirby's estimation to determine the *k*-values of the individuals,^15^ or using logistic regression,^53^ did not change the pattern of results (Supplementary Table S3). The proportion of immediate rewards chosen for each of the three groups was contrasted using a one-way ANOVA for all rewards, as well as a repeated-measures ANOVA to include the within-subject factor reward magnitude. As with the PIT task, significant findings (*P*<0.05) were followed by *post hoc t*-test analyses.

In addition, using Pearson correlations we investigated how closely related the behaviors of the participants in the two tasks were and, in an exploratory analysis, how mood was related to task performance.

## Results

The three groups did not differ in age, body mass index, years of education, trait impulsivity, BIS/BAS scores and affect intensity (one-way ANOVAs, all F(2,118)<1.86, *P*>0.16; Supplementary Table S4). Furthermore, participants were unaware whether they received one of the drugs or placebo, as assessed by post-experimental questionnaires (*χ*^2^(1)=1.00, *P*=0.32).

### PIT

**Figure 1:**
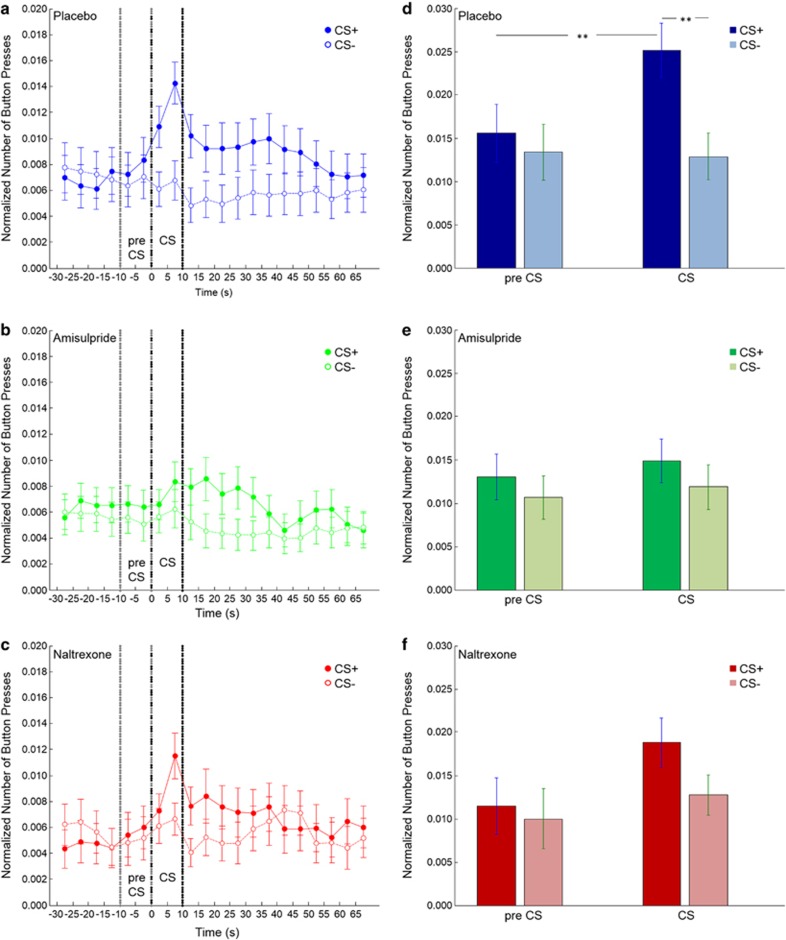
Button presses during the transfer-test phase of the Pavlovian-instrumental transfer task. (**a–c**) Button presses in 5-s bins before, during, and after presentation of the conditioned stimuli (CSs) for participants in the (**a**) placebo, (**b**) amisulpride and (**c**) naltrexone groups. The CS+ had previously been paired with chocolate; the CS− had not been paired with chocolate. The dotted lines indicate the pre-CS phase (−10 to 0 s) and the onset and offset of the CS phase (0–10 s). (**d–f**) The mean number of button presses in the pre-CS phase and the CS phase for participants in the (**d**) placebo, (**e**) amisulpride and (**f**) naltrexone groups (**P<0.005). The CS+ is displayed in dark and the CS− in light colors. Error bars represent s.e.m.'s.

To assess cue-induced responding, we compared the number of button presses during the transfer-test phase. Contrasting CS-induced button presses against pre-CS responding revealed a significant effect of time (F(1,104)=5.99, *P*<0.05). There was also a significant main effect of the CS type, with the rewarded CS increasing button presses in contrast to the unrewarded CS (F(1,104)=18.54, *P*<0.0001). Moreover, in line with a transfer effect, CS type interacted with time (F(1,104)=11.17, *P*<0.001), that is button presses increased specifically during the CS+ presentation. Importantly, we found a group*CS type*time interaction (F(2,104)=3.75, *P*<0.05), indicating that there were differences between our drug and placebo groups. As can be seen in Figure 1, in the placebo group button presses increased during the CS+ presentation as opposed to the 10 s before the CS presentation. Both drug groups showed less of an increase in button-pressing during the CS+ than the placebo group (Figures 1d–f). *Post hoc t*-tests revealed that for the placebo group the difference between button-pressing during the CS+ presentation was significantly higher than pre-CS+ presentation (*t*(37)=3.68, *P*<0.005), as well as significantly higher than during the CS− presentation (*t*(37)=5.35, *P*<0.001). This was not the case for the amisulpride and naltrexone groups (amisulpride: pre-CS+ versus CS+: *t*(34)=0.62, *P*=0.54; CS+ versus CS-: *t*(34)=1.66, *P*=0.11; naltrexone: pre-CS+ versus CS+: *t*(33)=1.92, *P*=0.06; CS+ versus CS-: *t*(33)=2.03, *P*=0.05). Furthermore, in both drug groups, the difference between button-pressing for the rewarded and unrewarded CSs during CS presentation was significantly reduced compared with the placebo group (amisulpride versus placebo: *t*(71)=3.01, *P*<0.01; naltrexone versus placebo: *t*(70)=2.13, *P*<0.05). There was no significant difference between the two drug groups (amisulpride versus naltrexone: *t*(67)=0.60, *P*=0.55). Thus, cue-induced responding was reduced by both amisulpride and naltrexone.

To assess whether the groups differed in how much they desired M&M's before or after the PIT task and in order to rule this out as a potential confound for subsequent analyses, we performed a repeated-measures ANOVA, which indicated that there was no significant main effect of group (F(2,104)=0.20, *P*=0.82). Thus, the drugs did not have an impact on desire for chocolate as such. Although the mean desire for chocolate across groups decreased from 83.9 (pre-test) to 67.3 (post-test), in all three groups it remained significantly larger than 50, the midpoint of the scale (placebo: *t*(37)=4.68, *P*<0.001; amisulpride: *t*(34)=3.10, *P*<0.01; naltrexone: *t*(33)=2.98, *P*<0.01).

In order to test whether the groups differed in their performance during the instrumental or Pavlovian phases, we also compared their responding and learning during these phases (Supplementary Table S2). Participants took on average 2.5 min (±0.22 s.e.m.) to complete the instrumental training and performed 113 (±0.86) button presses, or 1.33 (±0.08) button presses per second. There were no significant differences between the groups in the number of button presses (F(2,104)=0.85, *P*=0.43), the frequency of button presses (F(2,104)=0.08, *P*=0.92) or the time until criterion (F(2,104)=0.41, *P*=0.66). Similarly, in the Pavlovian acquisition phase, there were no significant differences between the groups in how well they learned the Pavlovian contingencies of the task (F(2,104)=2.08, *P*=0.13). Overall, it seems that, although the three groups did not differ in their desire for chocolate or their performance during the instrumental and Pavlovian acquisition phases, they differed in their behavior during the transfer-test phase. Thus, learning and desire was unaffected by the pharmacological manipulation, whereas cue-induced responding was reduced.

### Delay discounting

**Figure 2:**
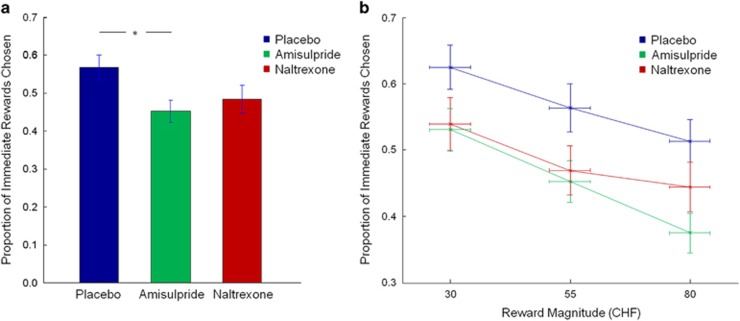
Proportion of smaller immediate rewards chosen in the delay-discounting task. (**a**) Participants in the amisulpride group chose significantly fewer smaller immediate rewards than those in the placebo group (*P<0.05). (**b**) Choice behavior of the different groups split by high, medium and large reward magnitudes. Vertical error bars represent s.e.m.'s proportion of immediate rewards chosen; horizontal error bars represent s.e.m.'s reward magnitudes. Higher values indicate higher reward impulsivity. CHF, Swiss Franks.

To test whether the dopamine and opioid receptor ligands affected reward impulsivity, we compared the performance of the three groups during the delay-discounting task. The groups differed significantly in the proportion of immediate rewards chosen (F(2,117)=3.18, *P*<0.05; Figure 2a). *Post hoc t*-tests revealed that the amisulpride group chose the smaller immediate rewards significantly less often than the placebo group (*t*(78)=2.58, *P*<0.01). The difference between the naltrexone and the placebo groups did not reach significance (*t*(78)=1.70, *P*=0.09). These data were largely the same when reward magnitude was included as an additional factor in the analysis. Again, we found a main effect of group (F(2,117)=3.18, *P*<0.05), but also a main effect of reward magnitude (F(2,116)=91.03, *P*<0.0001; Figure 2b), as well as a significant reward magnitude*group interaction (F(4,234)=2.44, *P*<0.05). *t*-tests indicated that the amisulpride group chose a lower proportion of immediate rewards than the placebo group for all reward magnitudes (small rewards: *t*(78)=2.02, *P*<0.05; medium rewards: *t*(78)=2.32, *P*<0.05; large rewards: *t*(78)=3.17, *P*<0.01). In contrast, although none of the comparisons reached significance, the difference between naltrexone and placebo participants was highest for small and medium rewards (small rewards: *t*(78)=1.65, *P*=0.102; medium rewards: *t*(78)=1.84, *P*=0.07; large rewards: *t*(78)=1.43, *P*=0.16). There were no significant differences between the two drug groups. Overall, it seems that both pharmacological manipulations led to a reduction in discounting, with the strongest effects for the amisulpride challenge and a nonsignificant trend for the naltrexone challenge.

### Relation between tasks

**Figure 3:**
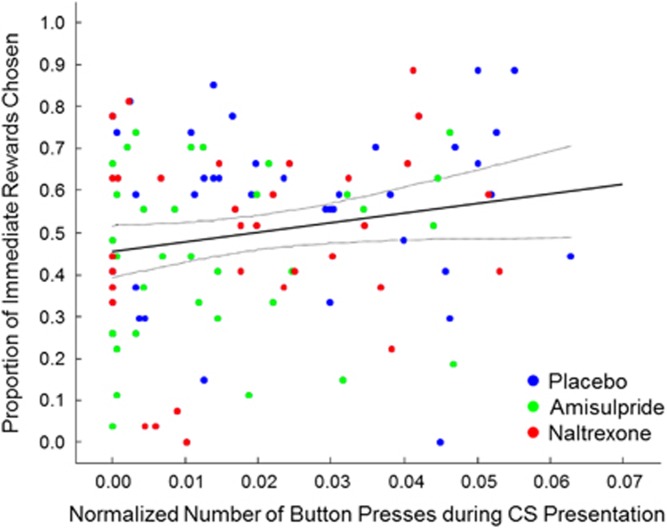
Absence of correlation between performance in the delay-discounting task and the Pavlovian-instrumental transfer task. Participants who choose more immediate rewards did not show a proportionate increase in button-pressing during the rewarded conditioned stimulus (CS) presentation (*r*=0.15, *P*=0.14, *N*=106). Placebo participants are displayed in blue, amisulpride participants in green and naltrexone participants in red.

Although the drugs elicited similar effects on both tasks, there was no significant correlation between the PIT effect and the proportion of immediate rewards chosen (*r*=0.15, *P*=0.14, *N*=106; Figure 3). Thus, the two tasks seem to measure different aspects of reward-guided behavior.

### Mood

Finally, in an exploratory analysis, we tested whether individual differences in mood might have influenced cue-induced responding and reward impulsivity. A one-way ANOVA revealed that the three groups differed in mood (F(2,116)=3.44, *P*<0.05). The mood of the amisulpride group was not significantly different from the mood of the naltrexone group (*t*(78)=0.56, *P*=0.58), but both drug groups showed lower mood ratings than the placebo group (placebo: 67.59 (±2.80 s.e.m.); amisulpride: 58.96 (±3.00 s.e.m.); naltrexone: 56.30 (±3.63 s.e.m.); amisulpride: *t*(77)=0.21, *P*<0.05; naltrexone: *t*(77)=0.25, *P*<0.05). We therefore re-performed all main analyses of group differences in cue-induced responding and reward impulsivity as analyses of covariance, using mood as a covariate, which produced similar results. In an exploratory correlation analysis we also investigated the influence of mood on our two behavioral tasks. There were no significant correlations between mood and behavioral outcomes in the PIT task; however, the impact of mood on delay discounting differed between the three groups. Whereas there was no correlation in the placebo group, elevated mood went along with a greater number of immediate rewards chosen in the amisulpride group (Supplementary Figure S3). In contrast, this relationship was reversed for the naltrexone group, where mood correlated negatively with the proportion of immediate rewards chosen. For statistics, please refer to the Supplementary results.

## Discussion

To our knowledge, this is the first study to contrast the effect of dopamine and opioid receptor blockade on PIT and delay discounting in healthy volunteers. Our data confirm the critical role of dopamine in *both cue-induced responding and reward impulsivity* in humans by showing that dopamine D2/D3 receptor blockade with amisulpride reduced the motivation to obtain immediate rewards in both a PIT task and a delay-discounting task. A blockade of μ- and κ-opioid receptors with naltrexone had similar albeit less pronounced effects on cue-induced responding, as well as a nonsignificant trend reduction in reward impulsivity. Although both substances reduced mood, they differently affected the relation between mood and delay discounting. Under amisulpride, increased reward impulsivity was correlated with positive mood, whereas in the naltrexone group it was associated with negative mood, suggesting that mood might be an important modulator of relapse risk under addiction treatment with dopamine and opioid antagonists.

### Cue-induced responding

We found that amisulpride reduced cue-induced responding as measured by PIT. These results concur with animal studies showing that an inactivation of the ventral tegmental area, which likely decreased dopaminergic activity in the nucleus accumbens, reduced PIT.^54, 55^ Moreover, systemic administration and microinjections in the nucleus accumbens of dopamine receptor antagonists impair the general form of PIT,^20, 22^ whereas intra-accumbal microinjections of the indirect dopamine agonist amphetamine facilitate general PIT.^30, 31^ Only a single human study has recently investigated the effects of a manipulation of the dopamine system on PIT: Hebart and Gläscher^21^ reported that a dietary depletion of the dopamine precursors tyrosine and phenylalanine reduces appetitive PIT, which is in line with our results. However, depletion of tyrosine/phenylalanine not only decreases dopamine but also noradrenaline synthesis^56^ and therefore the challenge has less specific effects on the dopamine system compared with the selective dopamine D2/D3 receptor antagonist amisulpride used in the present study.

The μ- and κ-opioid receptor antagonist naltrexone decreased PIT as well. This finding is in accordance with the report that both a stimulation of dopamine release by amphetamine as well as a stimulation of μ-opioid receptors by DAMGO microinjection in the nucleus accumbens increased cue-triggered levels of motivation to pursue sucrose reward in the PIT.^29^ Moreover, μ-opioid receptor knockout mice showed normal PIT, whereas δ-opioid receptor knockout mice were impaired. Similar effects were observed when μ- or δ-opioid receptor antagonists were injected into the nucleus accumbens.^28^ One human study has investigated opioid effects on cue reactivity in non-treatment-seeking alcoholics.^32^ The same dosage of naltrexone as used in the current study, over a 7-day period, produced no changes in craving, but led to a reduction in alcohol cue-induced neural activation in the ventral striatum. Our findings extend these results to healthy participants, separate the drug effect from the disorder effect and thereby provide a clearer picture of opioid effects on cue-induced responding

### Reward impulsivity

Our finding of reduced reward impulsivity under amisulpride is in line with previous animal studies showing that the indirect dopamine agonists amphetamine^34, 35^ and cocaine^36^ increase reward impulsivity, although also contradictory results exist.^33^ Moreover, one small human study (*n*=13) has also revealed increased reward impulsivity with indirect catecholamine agonism by L-DOPA (Pine *et al.;*^19^ but see De Wit *et al.*^17^ for opposing results with amphetamine, as well as Hamidovic *et al.*^37^ for null effects using oramipexole), but found no effect with the unselective dopamine antagonist haloperidol. Our results add to this literature by showing that selective blockade of D2/D3 receptors can reduce reward impulsivity.

Reward impulsivity was moderately reduced by naltrexone, although the reduction did not reach significance. Only few studies have investigated the effects of opioid challenges on reward impulsivity in humans and animals. For example, in one animal study the μ-opioid receptor agonist morphine dose-dependently increased reward impulsivity, whereas naltrexone alone did not affect the value of delayed rewards but blocked the effects of morphine.^25^ Two very small human studies showed no significant effects of naltrexone on reward impulsivity (nine abstinent alcoholics and nine healthy controls;^38^ nine abstinent alcoholics and ten healthy controls^57^). Interestingly, a PET study using a μ-opioid receptor-selective radiotracer revealed that individuals with high trait impulsivity showed elevated density of μ-opioid receptors in regions underpinning reward impulsivity, such as the nucleus accumbens and the amygdala.^26^

It is important to note that the primary effects of amisulpride and naltrexone on reward impulsivity, cue-induced responding and even mood were relatively similar. This is in line with the recently reported common involvement of the dopamine and the opioid system in the direct control of drug-‘*wanting*' behavior.^29^ On the other hand, naltrexone has been shown to block dopamine release in the nucleus accumbens, induced, for example, by alcohol^58^ or feeding.^59^ Indeed, the mesolimbic opioid and dopamine systems appear to be closely linked. For example, opiates inhibit GABAergic interneurons in the midbrain and thereby disinhibit dopamine neurons.^60, 61^ Consequently, naltrexone may have influenced behavior indirectly by a modulation of accumbal dopamine release. Invasive methods would be required to completely disentangle the direct from the dopamine-mediated impact of opioid receptor stimulation and blockade on reward impulsivity. However, the observation that the two drug challenges differentially affected the relation between mood and reward impulsivity is more in line with independent actions of naltrexone rather than actions that are mediated through an effect on dopamine neurons.

### Mood effects

On average, the mood of the amisulpride and of the naltrexone group was lower than the mood of the placebo group. This effect is plausible for naltrexone, for which dysphoria has been reported as a common side effect;^62^ however, the negative mood effect of amisulpride is surprising, given that the compound has been shown to be an effective antidepressant.^63^ Although these differences could not account for our findings when we included mood as a covariate, it is worth noting that more positive mood has previously been associated with increased reward impulsivity.^64^ Conversely, anhedonia is associated with reduced reward impulsivity^65^ and reduced willingness to exert effort for reward.^66^ More importantly, we found that both drug challenges exerted opposite effects on the relation between mood and reward impulsivity but had no effects on the relation between mood and cue-induced responding. This finding, together with the absence of a relation between cue-induced responding and reward impulsivity across the total study sample (Figure 3), suggests that cue reactivity and reward impulsivity may reflect distinct reward processes (see also Supplementary Discussion). It is conceivable that cue-induced responding is more strongly related to stimulus-induced value prediction, whereas reward impulsivity may reflect a bias of immediate rewards on the computation of decision value.

### Limitations

The following limitations should be kept in mind when considering our study. (1) Given that the PIT task cannot reasonably be repeated within an individual, we employed a between-subject design, although a within-subject design would have been advantageous regarding the reliability of the results. However, we aimed to compensate this limitation by investigating relatively large samples. (2) In order to maximize the number of subjects in each group, we only tested single doses of the two blockers. Varying the dosage may provide information about the relative influence of the dopamine and opioid systems on cue-induced responding and reward impulsivity. (3) Amisulpride blocks not only dopamine D2/3 receptors but also 5-HT7 receptors.^67^ In this regard it is worth noting that acute serotonin (tryptophan) depletion reduces reactivity to aversive cues, but has no effects on appetitive cues in general versions of PIT,^68^ which together with our results is in line with the notion that the dopamine and the serotonin systems have opposing roles in appetitive and aversive value processing. (4) The version of our PIT task does not allow to distinguish general forms of cue-induced responding from outcome-specific forms.^69, 70^ This permits only limited comparisons to animal studies that differentiate between these two types of PIT. (5) Our measure of mood as a single-item question at the end of the study provides only a global measure of mood state. Future studies should therefore apply a more sensitive measure of mood and measure baseline mood in order to confirm the relationship between mood and reward impulsivity and the modulatory effects of naltrexone.

## Conclusions

Although animal research provided promising findings,^71^ the efficacy of dopamine receptor antagonists for the treatment of addiction in humans appears to be limited.^9^ Our data suggest that it may be worth exploring the usefulness of the more specific D2/D3 dopamine receptor antagonist amisulpride, particularly in patients with increased reactivity to drug cues and elevated reward impulsivity. Moreover, it could be of interest to further explore the relationship of mood and reward impulsivity under naltrexone and amisulpride, as individual mood of the patient could potentially prove to be a relevant factor when deciding between treatment with amisulpride or naltrexone. In conclusion, we show that the opioid system contributes to increased responding to reward cues, whereas the effects on delay discounting were less pronounced in our study. In contrast, the dopamine system was involved in both responding to reward-associated cues and in delay discounting.

## Figures and Tables

**Table 1:** Human and selected animal studies investigating the role of dopamine and opioid in cue-induced responding and reward impulsivity

		*Substance*	*Dosage*	N	*Effect*	*Reference*
***CUE-INDUCED RESPONDING***						
	**Dopamine**					
Animal						
** **	D2/3 antagonist	Pimozide	0.25 mg kg^−1^ i.p.	32(B)	↓	^20^
		α-Flupenthixol	0.5 mg kg^−1^ i.p.	32(B)	↓	^20^
		Flupenthixol	0.5 mg kg^−1^ i.p.	24	↓	^23^
		Flupenthixol	0.05 and 0.25 mg kg^−1^ i.p.	14	↓(0.25 mg kg^−1^) ↔(0.05 mg kg^−1^)	^23^
		Flupenthixol	0.5 mg kg^−1^ i.p.	16	↓	^24^
		Raclopride	0.5- and 1.0 μg Intra-NAC	57(B)	↓	^22^
	D1 antagonist	SCH-23390	0.5- and 0.75 μg Intra-NAC	56(B)	↓	^22^
	Indirect DA agonist	Amphetamine	20 μg per 0.2 μl intra-NAC	45	↑	^29^
		Amphetamine	20 μg per 0.2 μl intra-NAC	14	↑	^30^
		Amphetamine	0.0, 2.0, 10.0 or 20.0 μg per 0.5 μl Intra-NAC	30	↑	^31^
Human						
	DA/NA depletion	Amino-acid mixture lacking TYR/PHE	90 g	69(B)	↓	^21^
						
	**Opioid**					
** **Animal						
** **	Mu-opioid receptor antagonist	CTAP	2 μg μl^−1^ Intra-NAC	48	↔	^28^
** **	Delta-opioid receptor antagonist	Naltrindole	5 μg μl^−1^ Intra-NAC	48	↓(For NAc shell) ↔(for Nac core)	^28^
** **	Mu-opioid receptor agonist	DAMGO	0.5 μg per 0.2 μl Intra-NAC	55	↑	^29^
** **Human						
** **	Unspecific opioid receptor antagonist	Naltrexone	50 mg p.o.	23(B)	↔(Craving) ↓(fMRI)	^32^

***REWARD IMPULSIVITY***						
	**Dopamine**					
** **Animal						
** **	D2/3 antagonist	Flupenthixol	0.5 mg kg^−1^ i.p.	8	↑	^18^
		Flupenthixol	25, 50 and 100 μg kg^−1^ i.p.	17	↓	^33^
		Haloperidol	0.01–0.1 mg kg^−1^ i.p.	24	↔	^34^
		Raclopride	40, 80 and 120 μg kg^−1^ i.p.	17	↓	^33^
** **	D1 antagonist	SCH-23390	5, 10 and 20 μg kg^−1^ i.p.	17	↔	^33^
** **	Indirect DA agonist	Amphetamine	0.5 and 1.0 mg kg^−1^ i.p.	17	↑	^33^
		d-Amphetamine	0.4–1.2 mg kg^−1^ s.c.	24	↑	^34^
		d-Amphetamine	0.25 and 0.5 mg kg^−1^ i.p.	8	↓(0.25 mg kg^−1^) ↔(0.5 mg kg^−1^)	^18^
		d-Amphetamine	0.80 and 1.20 mg kg^−1^ i.p.	24	↑	^35^
		Cocaine	15 mg kg^−1^ i.p.	5	↑	^36^
** **Human						
** **	D2/3 antagonist	Haloperidol	1.5 mg p.o.	13	↔	^19^
** **	D2/3 agonist	Oramipexole	0.25 and 0.5 mg p.o.	10	↔	^37^
** **	Indirect DA agonist	d-Amphetamine	10 mg or 20 mg p.o.	36	↓(20 mg) ↔(10 mg)	^17^
		L-dopa	150 mg p.o.	13	↑	^19^
						
	**Opioid**					
** **Animal						
** **	Unspecific opioid receptor antagonist	Naloxone	0.3, 1.0 and 3.0 mg kg^−1^ i.p.	16	↔	^27^
		Naltrexone	0.01, 0.1, 1.0 and 10 mg kg^−1^ s.c.	15	↔	^25^
** **	Mu-opioid receptor agonist	Morphine	0.3, 1.0, and 1.8 mg kg^−1^ s.c.	15	↑	^25^
		Morphine	0.3, 1.0, 3.0 and 6.0 mg kg^−1^ i.p.	16	↑(6.0 mg kg^−1^)	^27^
** **Human						
** **	Unspecific opioid receptor antagonist	Naltrexone	50 mg p.o.	18	↔	^38^

Abbreviations: DA, dopamine; fMRI, functional magnetic resonance imaging; i.p., intraperitoneal injection, intra-NAC, intra nucleus accumbens microinjections; N, number of subjects; NA, noradrenaline; p.o., per oral administration; s.c., subcutaneous injection; TYR/PHE, tyrosine/phenylalanine.

All studies are within-subject, unless marked ‘B' (between subject). Effects are abbreviated as: ↓= decrease,↔= no effect, ↑= increase.

As the present study focused on cue-induced responding and reward impulsivity in humans, only representative animal studies are listed. For a more exhaustive review please refer to Holmes *et al.*^13^ and Bari and Robbins.^39^

## Supplementary Material

Supplementary Informations
