# Supplementary material for: Dopamine D2/3- and μ-opioid receptor antagonists reduce cue-induced responding and reward impulsivity in humans
Source: Transl Psychiatry. 2016 Jul 5;6(7):e850–. doi: 10.1038/tp.2016.113 (PMC4969763; doi:10.1038/tp.2016.113)
Supplement: Supplementary Informations [file tp2016113x1.pdf]

Supplementary Material

Dopamine D2/3- and μ-opioid receptor antagonists reduce cue-induced responding and reward impulsivity in humans

Susanna C. Weber[1], Beatrice Beck-Schimmer[2], Marie-Elisabeth Kajdi[2], Daniel Müller[3], Philippe N. Tobler[1,4]*, Boris B. Quednow[4,5]*

[1]*Laboratory for Social and Neural Systems Research, Department of Economics, University of Zurich, 8006 Zurich, Switzerland*
[2]*Institute of Anesthesiology, University Hospital Zurich, Switzerland*
[3]*Institute of Clinical Chemistry, University Hospital Zurich, Switzerland*
[4]*Neuroscience Center Zurich, University of Zurich and Swiss Federal Institute of Technology Zurich, Switzerland*
[5]*Experimental and Clinical Pharmacopsychology, Department of Psychiatry, Psychotherapy and Psychosomatics, Psychiatric Hospital, University of Zurich, Switzerland*

*These authors contributed equally

Supplementary methods. Pavlovian-instrumental transfer task.

Supplementary results. Mood.

Supplementary discussion. Relation between tasks.

Figure S1. Timing of the behavioral tasks.

Figure S2. Illustration of the set-up of the Pavlovian-instrumental transfer task.

Figure S3. Correlations between mood ratings and the proportion of immediate rewards chosen in the delay discounting task.

Table S1. Final number of subjects used in each analysis.

Table S2. Performance of the placebo, amisulpride, and naltrexone groups during the instrumental and Pavlovian phase, as well as the extinction period.

Table S3. Delay discounting results using Kirby's k and logistic regression to estimate discounting.

Table S4. Demographic data and questionnaire data of the placebo, amisulpride, and naltrexone groups.

Supplementary references.

Supplementary methods

Pavlovian-instrumental transfer task

The Pavlovian-instrumental transfer (PIT) task probes the ability of Pavlovian stimuli to increase instrumental reward responding even in the absence of any rewards. Accordingly, it is used to investigate reward-related behaviors, such as eating, drinking and drug taking, which are triggered by cues associated with drug or non-drug rewards. Controlled by Cogent software, a Med Associates M&M dispenser [Model ENV-702, St. Albans, VT] delivered individual M&M chocolates into a small bowl easily accessible to participants (Figure S1). To prevent auditory conditioning to the sounds made by the dispenser, participants wore headphones emitting constant 72-dB white noise. Before and after the task, as well as at the end of the experiment, participants were asked to indicate their desire for chocolate on a visual analogue scale from 0 (not at all) to 100 (very much): "Please mark on the scale how much you would want to eat M&Ms right now".

The PIT task followed a standard design according to the protocol of Lovibond and Colagiuri,[1] with an instrumental conditioning phase, a Pavlovian conditioning phase, and a transfer-test phase. In the instrumental phase, participants were instructed to press the space bar on their keyboard to earn chocolates. They received the following instructions: "During the first part of the experiment, you can press the space bar to obtain chocolates. You will need to press the space bar multiple times to earn each chocolate. You can press the space bar as much or as little as you wish. When you receive chocolate, please eat it. You can start pressing the space bar as soon as these instructions disappear." We used a variable-ratio (VR) 10 schedule, where on average 10 button-presses of the space bar (range=5–15) were required before a chocolate was delivered. For the first three rewards, fixed ratio schedules 2, 4, and 6 were used to induce button-pressing in participants. During the delivery of every chocolate reward, the word "chocolate" appeared in the center of the screen for 1 second. Additionally,

for every button-press a small black square appeared in the center of the screen for 0.1 seconds. Any participant that had not obtained at least five rewards in the first 5 minutes of the task was informed that they might have to press the space bar more than once in order to earn chocolate. The instrumental-acquisition phase completed either when participants had obtained 12 rewards or once 10 minutes had passed. As in the previous study,[1] any participant who did not earn 12 rewards did not meet criterion for the following parts of the task and was excluded from further analysis. In total, two placebo subjects, six amisulpride subjects and six naltrexone subjects did not meet the criterion of the instrumental phase and were therefore excluded from the PIT analysis.

In the second phase, the Pavlovian phase, participants were instructed that they would see images on the computer screen and told not to press the space bar. The following instructions were displayed: "During the next part of the experiment, you will see some colored images and you may or may not receive chocolate. Please do not press the space bar during this part of the experiment." We used a differential-conditioning procedure. A red and a blue stimulus (counterbalanced) acted as the two conditioned stimuli (CS): CS+ and CS-. Every CS was presented 6 times for 10 seconds. The CS+ was always paired with the delivery of a chocolate reward, while the CS- was always presented with no outcome. The intertrial interval ranged from 15 to 35 seconds and trials were randomized such that no more than two trials of the same CS type were presented in a row.

In the final phase, the transfer-test phase, participants were instructed that they could now press the space bar again: "During the next part of the experiment, you may press the space bar again." In this phase, testing was carried out under instrumental and Pavlovian extinction, i.e., without any rewards. During the initial 2 minutes, instrumental extinction took place. Extinction was extended for another 30 seconds for any participants that pressed the space bar during the final 30 seconds of the extinction period. Once participants had stopped responding for the entire final 30-second period or 10 minutes

had passed, the transfer test began. During the transfer test, the CS+ and the CS- were presented for 10 seconds in random order, while button presses were recorded. The two CSs were then presented again in random order. The intertrial interval ranged from 90 to 110 seconds. After this phase, participants rated how often each of the two CSs was immediately followed by chocolate during the second phase of the experiment, using a scale from 0 (never) to 100 (always). The difference between the rating for the CS+ and the rating for the CS- served as an index of participants' awareness of the Pavlovian contingencies.

Supplementary Results

Mood

With respect to the exploratory correlation analysis we found that while there were no significant correlations between mood and behavioral outcomes in the PIT task ($|r|<0.18$, $p>0.30$, $N_{placebo}=38$, $N_{amisulpride}=35$, $N_{naltrexone}=34$), the impact of mood on delay discounting differed between the three groups. There was no correlation in the placebo group ($r=0.18$, $p=.28$, $N=39$), however, the amisulpride group showed a positive correlation between mood and the proportion of immediate rewards chosen in the delay discounting task ($r=0.31$, $p<.05$, $N=40$), while the naltrexone group showed a negative correlation ($r=-0.36$, $p<.05$, $N=40$). The comparison of the strength of the correlations between the three conditions using Fisher's Test to compare the correlation coefficients was significant for comparisons involving naltrexone (placebo vs. amisulpride: $p=.54$; placebo vs. naltrexone: $p<.05$; amisulpride vs. naltrexone: $p<.01$).

Supplementary discussion

Relation between tasks

One interesting finding of our study is that cue-induced responding, assessed by PIT, and reward impulsivity, measured by the delay discounting task, did not correlate. Furthermore, mood differentially modulated performance in these two tasks; specifically mood had no effect on performance in the PIT task, while it affected performance in the delay discounting task. This could suggest that the PIT and delay discounting tasks are not measuring the exact same process and this is noteworthy as it is commonly assumed that *incentive salience* (or *wanting*) measured through cue-induced responding in the PIT task can be equated to *decision value/utility* measured by reward impulsivity in the delay discounting task.[2,3] However, to our knowledge no study in humans has directly compared participants' behavior in these two tasks. Studies in humans have primarily used the Barratt Impulsiveness Scale (BIS-11) questionnaire to measure trait impulsivity and reported conflicting results. While Watson *et al.*[4] failed to find a correlation between trait impulsivity and the PIT transfer effect, Garofalo *et al.*[5] report that participants, who were sign trackers, i.e., participants who unlike goal trackers focus more on the conditioned stimulus than the reward, had stronger PIT transfer effects as well as higher levels of trait impulsivity. It is important to note, however, that impulsivity is not one unified construct. Instead it can be parsed into at least three components: (1) self-reported i.e., trait impulsivity (measured by BIS-11); (2) impulsive action (measured for example by the Stop Signal Task) and (3) impulsive choice (measured by delay discounting).[6] In line with this, one animal study investigating individual differences in cue-induced responding differentiated between impulsive action and impulsive choice, and found that sign trackers tend to show stronger cue-induced responding and impulsive action, but did not differ from goal trackers in impulsive choices in a delay discounting task.[7] Together, it may be that specifically impulsive choice differs from the *incentive salience*/*wanting* behavior measured by the PIT task and that

decision value/utility is not directly influenced by the same processes that enhance cue responding. However, one should keep in mind that the absence of a correlation does not in itself offer definitive proof of a dissociation between these two constructs, and it would be interesting to probe this further in future studies.

Supplementary figures

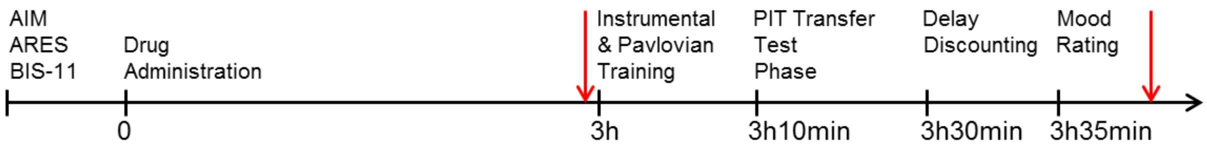

Figure S1. Timing of the behavioral tasks. After completing questionnaires, participants received 400 mg amisulpride, 50 mg naltrexone or placebo in a randomized and double-blind fashion. After 3h (+/- 1.10 min, SEM), participants underwent instrumental & Pavlovian training, followed by the PIT transfer test phase and the delay discounting task. Mood was assessed after completion of all tasks. Red arrows indicate blood plasma collection.

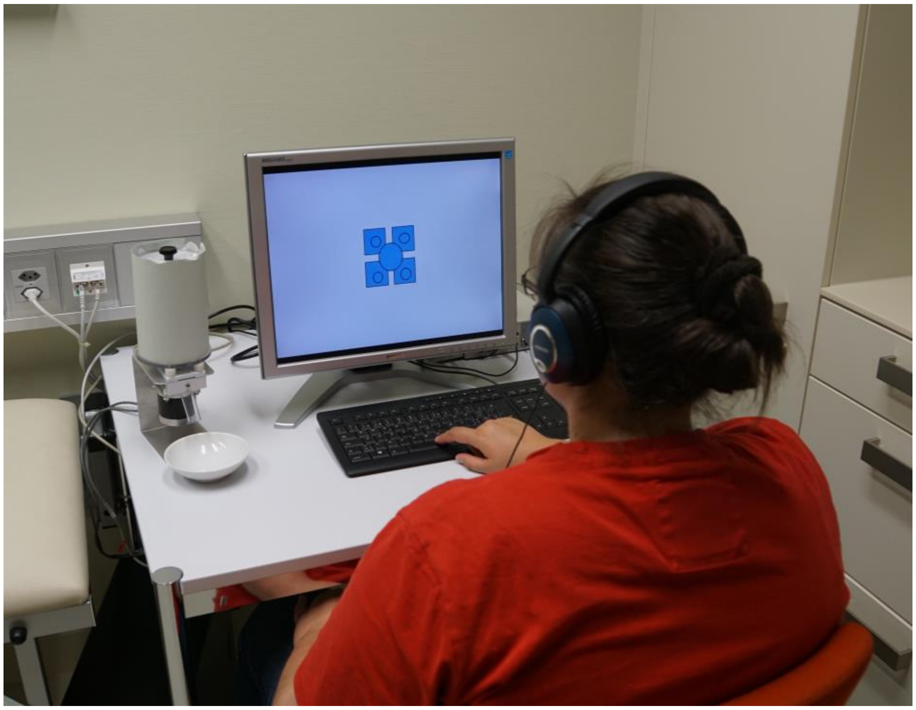

Figure S2. Illustration of the set-up of the Pavlovian-instrumental transfer task. Depiction of a participant during the transfer-test phase. A Pavlovian stimulus appears on the monitor, while the participant is pressing the instrumental key. During the prior instrumental and Pavlovian phases, M&Ms were dispensed into the white bowl to the left of the computer screen.

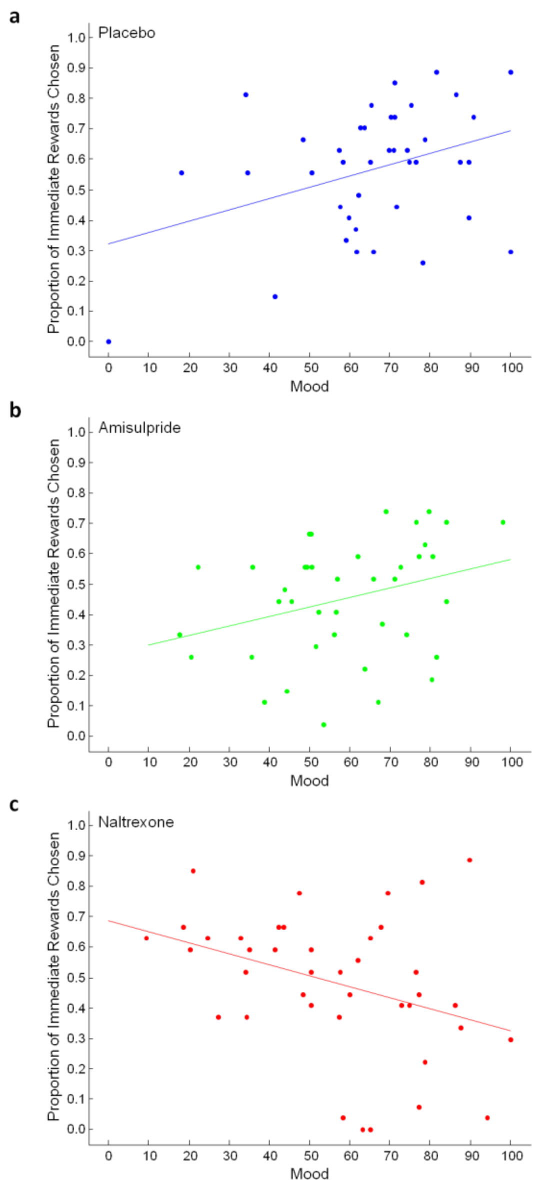

Figure S3. Correlations between mood ratings and the proportion of immediate rewards chosen in the delay discounting task. a-b The relationship of mood and discounting behavior was similar for participants in the **(a)** placebo and **(b)** amisulpride group, with mood and the number of immediate rewards chosen showing a positive relationship (placebo: r=.18, p=0.28, N=39; amisulpride: r=0.31, p<.05, N=40). **(c)** In contrast, for participants in the naltrexone group the relation was reversed, with positive mood being associated with higher propensity to choose delayed rewards (r=-0.36, p<.05, N=40).

Supplementary tables

Table S1. Final number of subjects used in each analysis.

	Placebo	Amisulpride	Naltrexone
	N	N	N
Demographic	40	41	40
PIT	38	35	34
DD	40	40	40
Mood	39	40	40
Mood correlation w/PIT	37	34	34
Mood correlation w/DD	39	40	40
Correlation between tasks	38	34	34

N number of subjects; *PIT* Pavlovian-instrumental transfer task; *DD* delay discounting task.

We excluded the following subjects: *PIT* → subjects who did not meet criterion; *DD* → one subject who had missing data; *Mood* → one placebo subject and one amisulpride subject with mood=zero (outlier); *Mood correlation PIT* → one subjects who did not meet criterion and subjects with mood=zero (outlier); *Mood correlation DD* → one amisulpride subject with missing data (and who had mood=zero) and one placebo subject with mood=zero (outlier); *Correlation tasks* → subjects who did not meet criterion for the PIT, one subject who had missing data in the DD.

Table S2. Performance of the placebo, amisulpride, and naltrexone groups during the Instrumental and Pavlovian phase, as well as the extinction period.

	Placebo (N=38)		Amisulpride (N=35)		Naltrexone (N=34)				
	Mean	SEM	Mean	SEM	Mean	SEM	F	df, df_{err}	p
Instrumental Phase									
Time until criterion (minutes)	2.73	0.47	2.25	0.31	2.41	0.34	0.41	2, 104	0.66
Number of Button-Presses	112.58	1.52	112.63	1.31	115.00	1.60	0.85	2, 104	0.43
Frequency of Button-Presses	1.37	0.14	1.30	0.14	1.30	0.14	0.08	2, 104	0.92
Pavlovian Phase									
Ratings of Contingencies	67.86	0.42	53.51	0.66	65.60	0.51	2.08	2, 104	0.13
Extinction Period									
Number of Button-Presses	9.19	1.08	10.53	1.06	9.39	1.08	0.45	2, 104	0.64

Table S3. Delay Discounting Results using Kirby's equation and logistic regression to estimate the discounting parameter k.

	Placebo (N=40)		Amisulpride (N=40)		Naltrexone (N=40)				
	Mean	SEM	Mean	SEM	Mean	SEM	F	df, df_{err}	p
Kirby's k	0.026	0.005	0.010	0.002	0.018	0.005	3.39	2, 117	0.04
Logistic k	0.026	0.006	0.011	0.002	0.020	0.005	2.91	2, 117	0.05

k indicates the discount rate parameter for which the value of the smaller immediate reward is equal to that of the larger delayed reward (indifference).

Table S4. Demographic data and questionnaire data of the placebo, amisulpride, and naltrexone groups.

	Placebo (N=40)		Amisulpride (N=41)		Naltrexone (N=40)				
	Mean	SEM	Mean	SEM	Mean	SEM	F	df, df_{err}	p
Age	22.15	0.38	21.46	0.38	21.65	0.33	0.96	2, 118	0.39
Body mass index	22.51	0.38	21.69	0.40	21.80	0.38	1.37	2, 118	0.26
Years of education	14.90	0.35	14.35	0.31	14.35	0.31	0.73	2, 118	0.48
Affect Intensity Measure[8]	3.33	0.05	3.36	0.06	3.45	0.87	0.92	2, 118	0.40
Action Regulating Emotion Systems[9]	54.09	1.02	55.15	0.92	54.80	1.05	0.29	2, 118	0.75
Behavioral Inhibition System (BIS)	21.81	1.03	22.71	0.77	22.58	0.95	0.27	2, 118	0.76
Behavioral Activation System (BAS)	32.28	0.42	32.44	0.41	32.23	0.48	0.07	2, 118	0.94
Barratt Impulsiveness Scale[10]	68.50	1.10	67.90	1.12	65.52	1.24	1.86	2, 118	0.16

Supplementary references

1 Lovibond PF, Colagiuri B. Facilitation of voluntary goal-directed action by reward cues. *Psychol Sci* 2013; **24**: 2030–37.

2 Berridge KC, Aldridge JW. Decision utility, the brain, and pursuit of hedonic goals. *Soc Cogn* 2008; **26**: 621–46.

3 Monterosso J, Piray P, Luo S. Neuroeconomics and the study of addiction. *Biol Psychiatry* 2012; **72**: 107–12.

4 Watson P, Wiers RW, Hommel B, de Wit S. Working for food you don't desire. Cues interfere with goal-directed food-seeking. *Appetite* 2014; **79**: 139–48.

5 Garofalo S, Di Pellegrino G. Individual differences in the influence of task-irrelevant Pavlovian cues on human behavior. *Front Behav Neurosci* 2015; **9**: 163.

6 Broos N, Schmaal L, Wiskerke J, Kostelijk L, Lam T, Stoop N *et al.* The relationship between impulsive choice and impulsive action: a cross-species translational study. *PLoS ONE* 2012; **7**: e36781.

7 Lovic V, Saunders BT, Yager LM, Robinson TE. Rats prone to attribute incentive salience to reward cues are also prone to impulsive action. *Behav Brain Res* 2011; **223**: 255–61.

8 Larsen RJ, Diener E. Affect intensity as an individual difference characteristic: A review. *Journal of Research in personality* 1987; **21**: 1–39.

9 Hartig J, Moosbrugger H. Die “ARES-Skalen” zur Erfassung der individuellen BIS-und BAS-Sensitivität. *Zeitschrift für Differentielle und Diagnostische Psychologie* 2003; **24**: 293–310.

10 Patton JH, Stanford MS, Barratt ES. Factor structure of the Barratt impulsiveness scale. *J Clin Psychol* 1995; **51**: 768–74.
